# The yield and usefulness of PAIN^+^ and PubMed databases for accessing research evidence on pain management: a randomized crossover trial

**DOI:** 10.1186/s40945-021-00100-7

**Published:** 2021-04-01

**Authors:** Vanitha Arumugam, Joy C. MacDermid, Dave Walton, Ruby Grewal

**Affiliations:** 1 Physiotherapist, St. Joseph’s Health Centre, London, Ontario Canada; 2 Hand and Upper Limb Centre Clinical Research Laboratory, St. Joseph’s Health Centre, London, Ontario Canada; 3 School of Rehabilitation Science, McMaster University, Hamilton, Ontario Canada; 4 School of Physical Therapy, University of Western Ontario, London, Ontario Canada

**Keywords:** PAIN^+^, PubMed, Abstract coding, Descriptive classification, Preference, Perceived usefulness

## Abstract

**Introduction:**

PAIN^+^ and PubMed are two electronic databases with two different mechanisms of evidence retrieval. PubMed is used to “Pull” evidence where clinicians can enter search terms to find answers while PAIN^+^ is a newly developed evidence repository where along with “Pull” service there is a “Push” service that alerts users about new research and the associated quality ratings, based on the individual preferences for content and altering criteria.

**Purpose:**

The primary purpose of the study was to compare yield and usefulness of PubMed and PAIN^+^ in retrieving evidence to address clinical research questions on pain management. The secondary purpose of the study was to identify what search terms and methods were used by clinicians to target pain research.

**Study design:**

Two-phase double blinded randomized crossover trial.

**Methods:**

Clinicians (*n* = 76) who were exposed to PAIN^+^ for at least 1 year took part in this study. Participants were required to search for evidence 2 clinical question scenarios independently. The first clinical question was provided to all participants and thus, was multi-disciplinary. Participants were randomly assigned to search for evidence on their clinical question using either PAIN^+^ or PubMed through the electronic interface. Upon completion of the search with one search engine, they were crossed over to the other search engine. A similar process was done for a second scenario that was discipline-specific. The yield was calculated using number of retrieved articles presented to participants and usefulness was evaluated using a series of Likert scale questions embedded in the testing.

**Results:**

*Multidisciplinary scenario:* Overall, the participants had an overall one-page yield of 715 articles for PAIN^+^ and 1135 articles for PubMed. The topmost article retrieved by PAIN^+^ was rated as more useful (*p* = 0.001). While, the topmost article retrieved by PubMed was rated as consistent with current clinical practice (*p* = 0.02). PubMed (48%) was preferred over PAIN^+^ (39%) to perform multidisciplinary search (*p* = 0.02).

*Discipline specific scenario:* The participants had an overall one-page yield of 1046 articles for PAIN^+^ and 1398 articles for PubMed. The topmost article retrieved by PAIN^+^ was rated as more useful (*p* = 0.001) and consistent with current clinical practice (*p* = 0.02) than the articles retrieved by PubMed. PAIN^+^ (52%) was preferred over PubMed (29%) to perform discipline specific search.

**Conclusion:**

Clinicians from different disciplines find both PAIN^+^ and PubMed useful for retrieving research studies to address clinical questions about pain management. Greater preferences and perceived usefulness of the top 3 retrieved papers was observed for PAIN^+^, but other dimensions of usefulness did not consistently favor either search engine.

**Trial registration:**

Registered with ClinicalTrials.gov Identifier: NCT01348802, Date: May 5, 2011.

## Introduction

Pain is arguably the most common reason for people presenting to a clinician. Multiple disciplines are involved in pain management including physicians, nurses, physical therapists, occupational therapists and psychologists, with each discipline having specific roles and perspectives [1]. Thus, clinical questions about pain, are likely to be a shared concerns, across professions.

Evidence-based practice (EBP) is commonly regarded as a method for applying the best research to answer clinical questions [2]. Five steps are involved in successful implementation of EBP: step 1 is to formulate an answerable question; step 2 involves tracking down the best evidence; step 3 involves critical appraisal of the evidence retrieved; step 4 involves application of the evidence to the individual and step 5 is to assess the outcome of the process and make changes as necessary [3]. Accessing and appraising evidence has been one of the most consistent barriers to EBP across professions [4–6]. Accordingly, one of the most substantial developments supporting EBP has been the evolution of methods that support evidence retrieval and appraisal. With the ever-increasing proliferation of research evidence, electronic databases and strategies for extracting relevant research from those databases are critical components of optimizing EBP.

Electronic databases are repositories that hold a wealth of information. To achieve the expected benefits of using EBP clinicians must be able to “pull” the highest quality research from the evidence pool to address specific clinical research questions. A recent alternative approach has been to off-load. Databases like PubMed, SCOPUS, EMBASE etc. fall under the PULL category. More recently, strategies have been developed to “push” research out to target users. Where the evidence can be targeted and customized to the end user, greater uptake might be anticipated.

The most commonly searched electronic database for pain research evidence is PubMed [7]. PubMed is a service of the US National Library of Medicine® that provides free access to MEDLINE®, the NLM® database of indexed citations and abstracts of medical, nursing, dental, veterinary, health care, and preclinical sciences journal articles. As of April 2018, there were 5235 journals were indexed by MEDLINE. PubMed also indexes a selected set of life sciences journals not in MEDLINE. The usefulness of PubMed in retrieving evidence has been tested in different areas of medical practice [8–14] but not with respect to pain evidence. PubMed facilitates improves access to research evidence by providing a comprehensive platform that can be searched for answers to specific clinical research questions. However, the results are typically presented chronologically, not according to quality.

PAIN^**+**^ (Premium LiteratUre Service) is an electronic evidence service created by Dr. Joy MacDermid, in collaboration with the Health Information Research Unit (HIRU), at McMaster University who developed the platform for the McMaster Premium Literature Service, (McMasterPLUS™) [15] and provides the technical expertise and infrastructure to support multiple evidence repositories and “pull” (retrieval) and “push” (alerting) services. PAIN^+^ is now called “PAIN+ CPN” (where CPN stands for Chronic Pain Network). Within the context of this study we will use the term PAIN^+^, the way it was called when the study was completed. PAIN^+^ was designed to provide access to pre-appraised current best evidence on pain to support clinical decisions. At the time the study was conducted, it included 110 premier clinical journals that address pain. All the citations from these journals are pre-rated for evidence quality by research staff and then clinical relevance and interest are rated by at least 3 members of an international panel comprising of clinicians (physicians, nurses, physical and occupational therapists, and clinical psychologists) with a common interest in pain management. PAIN^**+**^ facilitates the second and third step of EBP model, (retrieving evidence and rapid appraisal of quality of the evidence). PAIN^+^ falls under the PUSH category since alerts can be sent out to clinicians according to their preferences. Unlike PubMed it does not attempt to be comprehensive, but rather is selective about what is included in the database.

Clearly these two types of evidence repositories differ. PubMed provides a much broader scope of literature but does not evaluate the quality of the individual articles. Depending on the search strategy there is potential for a larger number of papers to be retrieved, but the relevance may be questionable when high volumes of research are retrieved. PAIN^+^ was designed to focus on the most relevant pain research and to provide targeted high-quality studies to practitioners interested in pain management. Because the extraction and quality appraisal process is labor-intensive, the number of journals abstracted is limited to those that provide a consistent yield of pain related research. Hence, PAIN^+^ may miss important pain studies published in journals not targeted for extraction, because pain is not a common focus. Due to these differences it is important to understand how these two different approaches perform when addressing clinical questions on pain management. In the current study, the focus is on the “PULL” aspect of the PAIN^+^.

The primary purpose of the study was to compare yield and usefulness of PubMed and PAIN^+^ in retrieving evidence to address clinical research questions on pain management. The secondary purpose of the study was to identify what search terms and methods were used by clinicians to target pain research.

## Methods

### Participants

One hundred twenty PAIN^**+**^ users [30 Physicians (MDs), 30 Registered Nurses (RN), 30 Occupational Therapist (OTs)/ Physical Therapists (PTs), 30 Psychologists (Psychs)] who have been exposed to PAIN^**+**^ for more than 1 year were invited via email to participate in this study. This study was completed between 2013 and 2015. We included a multidisciplinary group as pain management has moved from a discipline-specific approach to a broader multi-disciplinary approach. Of the 120 health care professionals who were invited, 76 agreed to be part of the study and were enrolled in the current study. Sample characteristics are described in Table 1. The study was approved by McMaster Research Ethics Board, Hamilton, Ontario, Canada.

**Table 1:** Demographics and search characteristics

Sample Characteristics n (%)	
n	77 (100)
Physicians	12 (16)
Nurses	27 (35)
Physiotherapists	21 (27)
Occupational Therapists	10 (13)
Psychologists	7 (10)
Search Characteristics	
Number of professionals who used Boolean searches	27
Number of unique search terms used	45
Total number of searches performed	308
Number of original research articles retrieved	
Total	4294
PubMed – multidisciplinary	1135
PAIN^+^ − multidisciplinary	715
PubMed - discipline-specific	1398
PAIN^+^ − discipline-specific	1046
Preference for electronic database in % (PubMed: PAIN^+^:No preference) *p* = 0.02	
For multidisciplinary scenario	48: 39: 13
For discipline-specific scenario	29: 52: 19

### Study design

Two-phase, double blinded randomized crossover trial.

#### Randomisation

Once the participants were recruited for the study, they were randomly allocated to the PAIN^**+**^ arm or the PubMed arm by an algorithm within the PAIN^**+**^ platform.

#### Allocation concealment

The study was completed in an online environment within the PAIN^**+**^ platform. An algorithm within the PAIN^**+**^ system would automatically assign the participants to either PAIN^+^ or PubMed randomly. Thus, generating a random order for allocation and also ensures concealment until assignment automatically. Hence there was no selection bias during intervention assignment.

#### Blinding

We used double blinding where the participants and the research assistants who collected the data were blinded to the allocation. This study was conducted using the PAIN^**+**^ platform hence the name of the database was still on the interface (See Appendix 2). However, the search yield from PAIN^+^ and PubMed looked similar and without any indication as to which search engine yielded those results, we still were able to ensure blinding until the data collection is complete.

#### Interventions

Participants were asked to perform a literature search for a multi-disciplinary and discipline-specific clinical question. Participants entered their search terms into an user interface (these were retained for analysis). Behind the interface, either the PubMed or PAIN^+^ databases operationalized the search, and the yields were presented within the interface back to participants.

#### Wash-out period

Participants crossed over to the second search engine immediately, without any wash-out period.

### Outcome measures

#### Primary outcomes

The primary outcomes were search yield from the outputs, and usefulness indicators rated by participants after each retrieval. (See Appendix 1).

### Search yield indicators

#### One-page yield

For each participant, the individual first-page retrieval was calculated as one-page yield. It was the total number of articles that were presented on the first page of the retrieval, up to a maximum of 20 articles, to reflect the typical number of citations that would be presented on a first-page retrieval.

#### Overall one-page yield

This was calculated by adding up one-page yield of all the participants.

### Usefulness indicators

Likert scales, embedded in sessions, were used to measure the different dimensions of the usefulness of PubMed and PAIN^**+**^ in retrieving pain evidence. Questions were presented at the end of each scenario. The questions focused on the following areas: usefulness of retrieved evidence, relevancy to practice, quality of the retrieved evidence, potential ability of the evidence to change practice, usefulness of the search session and rating of search engines (See Appendix 1).

#### Secondary outcomes

Our secondary outcomes were the different search terms used and their frequency. We also collected information on whether the participants used Boolean operators or not.

### Procedure

**Fig. 1:**
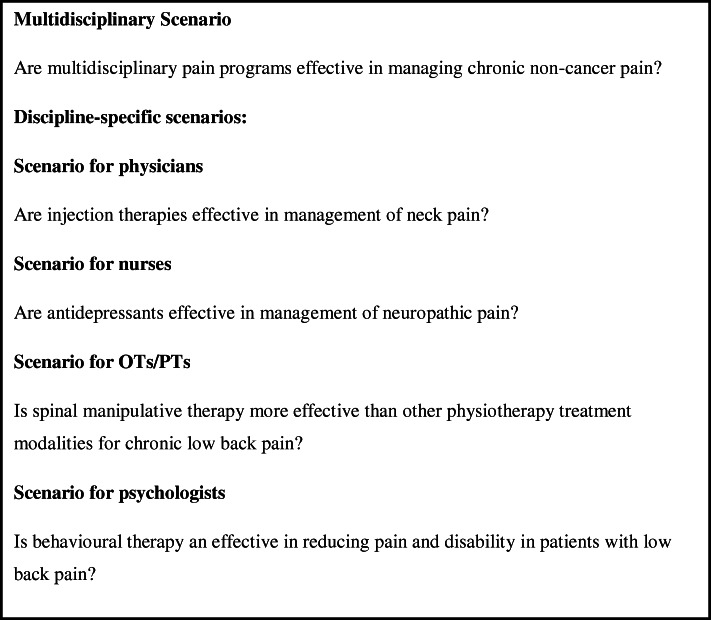
Clinical questions presented to clinicians in the study

**Fig. 2:**
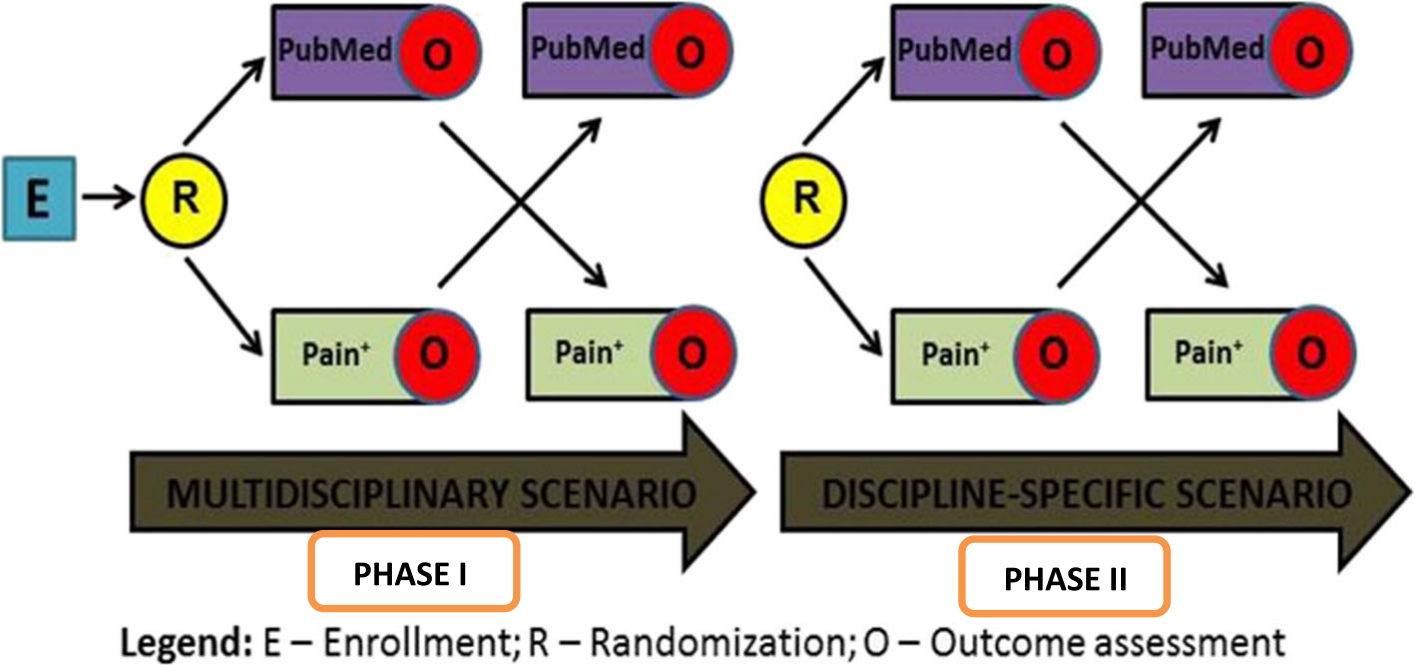
Crossover study design

Participants interacted with the study scenarios (See Fig. 1) through a single electronic interface within the PAIN^**+**^ platform (See Appendix 2) that presented two clinical questions. This interface was a single search window, with no additional search/syntax options. There were no special instructions provided regarding the use of Boolean operators. However, the clinicians were free to use Boolean operators. In the first query, clinicians were presented with a multi-disciplinary pain-related research question (identical for all study participants) “Are multidisciplinary pain programs effective in managing chronic non-cancer pain?” and in the second part, they were presented with a discipline-specific clinical question (See Fig. 1). Participants were asked to perform a literature search to find articles relevant to the presented clinical scenario. Behind the interface, the search was being conducted either in PubMed or PAIN^+^ depending on their allocation. Participants picked their keywords and could do one revised search if no citations were retrieved in the initial search (See Fig. 2).

Once the search terms were entered, participants were presented with the first 20 articles retrieved from the search. The results did not include any other details other than the title. If they click on the title, they can read the abstract. The information presented for both the search engines were the same and it is not possible to tell the difference. Results were presented based on the default for that search engine at the time of the study and could not be changed by the users. For PAIN+ it was the “best match” and for PubMed it was based on “most recent”. In addition, for PAIN+, no ratings or comments were provided. The participants were asked to select the top 3 relevant articles (without having to read the abstract). They were then asked to pick their top paper out of the 3 relevant papers and read the abstract. Then they responded to the following questions: *‘How would you rate the quality of this paper?’* (5-Point Ordinal scale); ‘*Based on the information in this paper, how likely would you change your practice?’* (7- point Likert scale). Then there were asked “Is the conclusion of this study consistent with your current practice?” and were asked to respond yes/No. They rated the usefulness of each of the 3 citations selected and then the usefulness of the overall session on a 7-point Likert scale (See Appendix 1).

After the completion of the search in the initial arm, the participants crossed over to the other search engine (PubMed or PAIN^**+**^). We did not include a wash-out period in the study. They were again asked to repeat their literature search and find articles relevant to the presented clinical scenario through the interface which provided output from the alternative database. After both searches were completed for the multidisciplinary scenario, participants were asked the last question: *Which search was better “Search1 or Search 2”?* Participants then moved on to the discipline-specific scenario (Phase-II where they were presented with a second discipline-specific pain-related research question. The scenarios were created to represent interventions that were relevant for the different professions. Thus, for the second scenario patients responded to a clinical question that was aligned with their professional background (See Fig. 1). The process that was followed in phase-I was repeated.

## Statistical analysis

Descriptive statistics and all the analyses were completed using SPSS version 22.^1^ Statistical significance was set at a level of *p* < 0.05. Independent t-tests were used to compare respondent’s ratings of PubMed and PAIN^**+**^ for all the usefulness questions. The response to the question” Is the conclusion of this study consistent with your current practice?” Was evaluated using the Chi-square test.

## Results

### Participants

Out of the 120 health care professionals who were invited, 76 agreed to be part of the study and were enrolled in the study. The majority of the sample was comprised of nurses (*n* = 27), while the smallest subgroup was the 7 psychologists (See Table 1).

On the whole 308 searches were made and 4294 articles were retrieved (See Table 1). For the multidisciplinary scenario, 77 searches on PubMed retrieved 1135 articles while a similar number of searches in PAIN^+^ retrieved 715 articles. For the discipline-specific scenario, PubMed yielded 1398 articles while PAIN^+^ retrieved 1046 articles. (See Table 1).

### Usefulness of search engines

#### Multidisciplinary scenario

The top 3 articles retrieved by PAIN^+^ (Mean 5.15; SD 1.13) were rated as more useful than the top 3 articles retrieved by PubMed (Mean 4.05; SD 1.55) (Mean difference = 1.10; 95% CI 0.66–1.54; *P* < 0.001). For the dimension, consistency of the most relevant citation with current clinical practice, it was found that PubMed (56%) was rated higher than PAIN^+^ (37%) (Chi-square value 11.92; *p* < 0.001) (See Table 2) There was no statistically significant difference between the two search engines in how clinicians rated the following usefulness criteria: quality of the most relevant paper; change of practice in the future; and usefulness of the overall session (See Table 3). Participants preferred PubMed (48%) over PAIN^+^ (39%) (Chi-square = 13.82; *p* < 0.001) to conduct searches for this type of question (See Table 1).

**Table 2:** Chi-square test of independence to compare the consistency of the results with clinical practice retrieved from PAIN^+^ and PubMed

*n* = 77	Percentage who agreed – PAIN^+^	Percentage who agreed – PubMed	Pearson Chi-square	Degrees of freedom	*P* value
Multidisciplinary scenario	37%	56%	45.64	4	0.001*
Discipline-specific scenario	65%	51%	11.92	4	0.02*

*significant at *p* < 0.05

**Table 3:** Independent t test for effectiveness of PAIN^+^ Vs PubMed in retrieving evidence on pain for a multidisciplinary scenario

Effectiveness characteristic	Electronic database	N	Mean	SD	T value	***P*** value
**Usefulness rating of Top 3** [1- Not useful at all to 7- Very useful]	PAIN^+^	77	5.15	1.13	4.97	0.00*
	PubMed	77	4.05	1.55		
**Rate the quality of paper** [1Very Low – 5 Very High]	PAIN^+^	77	3.69	0.70	1.87	0.06
	PubMed	77	3.51	0.70		
**Change practice based on the article retrieved** [1- Not likely at all to 7- Very likely]	PAIN^+^	77	1.61	0.60	−1.90	0.06
	PubMed	77	1.75	0.56		
**Usefulness of the overall session** [1- Not useful at all to 7- Very useful]	PAIN^+^	77	4.31	1.74	0.52	0.60
	PubMed	77	4.22	1.65		

*significant at *p* < 0.05

#### Discipline-specific scenario

The top 3 articles retrieved by PAIN^+^ (Mean 5.54; SD 1.38) were rated as more useful than the top three articles retrieved by PubMed (Mean 4.91; SD 1.50) (Mean difference = 0.63; 95% CI 0.21–1.04; *P* < 0.004) (See Table 4). When comparing the consistency of the results of the most relevant study with current clinical practice, PAIN^+^ (65%) was rated higher than PubMed (51%) (Chi-square value 45.63; *p* < 0.02) (See Table 2). Clinicians reported that they were more likely to change practice in the future based on the evidence retrieved by PubMed (Mean 1.58; SD 0.62) when compared to PAIN^+^ (Mean 1.36; SD 0.49) (mean difference = 0.22; 95% CI 0.08–0.35; *p* < 0.002). The quality of the most relevant paper; and overall usefulness of the session were not statistically different between the two search engines (See Table 3). Participants preferred PAIN^+^ (52%) over PubMed (29%) (Chi-square = 12.96; *p* < 0.002) (See Table 1).

**Table 4:** Independent t test for effectiveness of PAIN^+^ and PubMed in retrieving evidence on pain for discipline-specific scenarios

Effectiveness characteristic	Electronic database	N	Mean	SD	T value	***P*** value
**Usefulness rating of Top 3** [1- Not useful at all to 7- Very useful]	PAIN^+^	77	5.54	1.38	2.99	0.00*
	PubMed	77	4.91	1.50		
**Rate the quality of paper** [1Very Low – 5 Very High]	PAIN^+^	77	4.03	0.78	1.22	0.22
	PubMed	77	3.92	0.75		
**Change practice based on the article retrieved** [1- Not likely at all to 7- Very likely]	PAIN^+^	77	1.36	0.49	−3.23	0.00*
	PubMed	77	1.58	0.62		
**Usefulness of the overall session** [1- Not useful at all to 7- Very useful]	PAIN^+^	77	5.07	1.78	1.91	0.059
	PubMed	77	4.62	1.88		

*significant at *p* < 0.05

#### Description of the search terms and use of Boolean operators

**Fig. 3:**
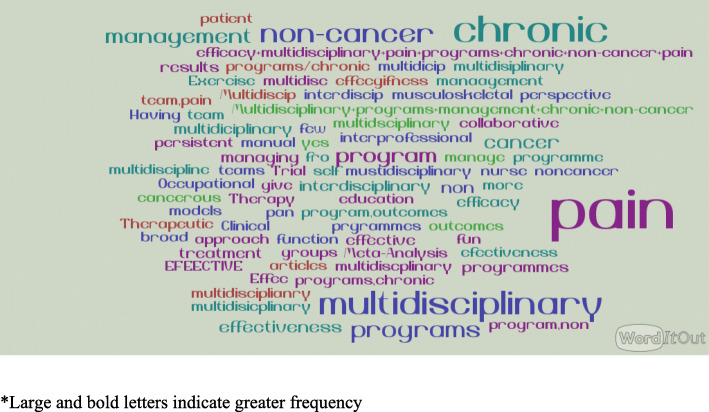
Word cloud depicting the search terms used for searches by the participants of the study for multidiscipline specific query

**Fig. 4:**
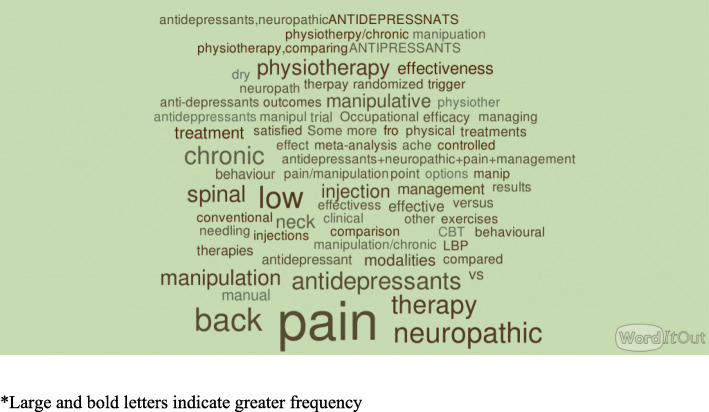
Word cloud depicting the search terms used for searches by the participants of the study for discipline specific query

Boolean operators such as the use of “AND”, “OR” and “NOT” to connect search terms were used by 29% of the participants. Over all 45 unique terms were used by our participants for their search. The top 10 search terms are listed in Table 5. The search terms used for a multidisciplinary query (see Fig. 3) and discipline-specific queries (see Fig. 4) are shown in a word cloud generated using NVivo software.^2^ Interpretation of a word cloud is as follows: The larger the word, the more frequently it was mentioned. As such it provides a visual of the search terms used by the participants.

**Table 5:** Frequently used search terms

Search Terms	Frequency
non-cancer	75
cancer pain	61
multidisciplinary pain	46
pain programs	41
chronic pain	39
pain program	21
pain treatment	4
pain programs	3
meta-analysis	3
clinical trial	3

## Discussion

The results of the current study indicate that both PubMed and PAIN^+^ retrieved useful pain research in both a multi-disciplinary and discipline-specific context. Although findings were inconsistent across some study measures and scenarios, overall participants preferred PAIN^+^ to a greater extent and found the top 3 papers to be more clinically useful.

The participants in our study noted that the top 3 articles that were retrieved by PAIN^+^ were more useful than PubMed for both multidisciplinary and discipline-specific queries. The reasons for this could be that PAIN^+^ is designed to identify, appraise and push out the most relevant high-quality research. Experts evaluate the quality of the papers that are considered for inclusion in PAIN^+^ and it might be that higher quality papers are considered more useful by practitioners. However, the second component of the PAIN^+^ evaluations is done by clinicians to evaluate how newsworthy and relevant the retrieved searches were. This latter step would certainly also be expected to contribute to the inclusion of more relevant research evidence in PAIN^+^. This supports the investment in time and effort by program administrators and clinicians to produce these ratings. Since PubMed has broad coverage and does not filter out studies based on quality unless instructed to do so, simplistic searches may yield studies that include lower quality research papers or that are less specifically focused on pain. This may account for the user perspective that the top three papers overall were less useful. There are strategies that can be used within PubMed to avoid this limitation such as manual use of search delimitators. Clinical queries is one such option within PubMed, which has built-in customized search filters, that help focus the retrieval on different types of clinical research questions or study designs [16–22]. These filters provide more targeted results than user-generated search terms [23]. The importance of these filters or customized search platforms like PAIN^+^ is confirmed by our finding that relatively unsophisticated search strategies (limited use of Boolean operators) were used by most users and that few exercised the options to manually focus their search strategies to better quality studies.

The quality rating of the topmost article retrieved, similar trends were observed in both multi-disciplinary and discipline specific queries. PAIN^+^ was rated higher than PubMed; however, the trend was not statistically significant. The reasons why the difference did not reach statistical significance could be that only the topmost article was rated for quality; and this may not reflect the quality of the overall one-page yield, particularly since most users would be expected to review more than the first paper in a one-page yield. Future studies should allow users to rate the quality of at least the top 5 abstracts which can be a better indicator of the quality of the overall yield. In addition, the fact that the quality ratings were based on the abstracts and not based on the full text might have affected the quality ratings. Also, another trend that was observed for both the searches was that usefulness was rated higher than quality; however, the difference in quality did not achieve the threshold for statistical significance. Further research is important to understand the perceptions of clinicians towards the quality and usefulness of health-related research and the perceived factors that drive these conclusions.

Our study indicated that clinicians preferred PubMed (48%) over PAIN^+^ (39%) for multidisciplinary scenario, (*p* < 0.005) while PAIN^+^ (52%) was preferred over PubMed (29%) for discipline specific searches (*p* < 0.003). However, it should be noted that the difference in the percentage of clinicians who preferred PubMed over PAIN^+^ for multidisciplinary queries was only 9% when compared to a 23% difference in clinicians who preferred PAIN^+^ over PubMed for discipline specific queries. The reasons why clinicians prefer one search engine is unclear and could relate to the number or quality of the evidence; or the extent to which it aligned with an individual clinician’s interests or practice patterns. However, since the “front face” of the search was uniform across all searches, this could not have been a factor.

The articles retrieved by PAIN^+^ were rated as more consistent with current practice and those retrieved by PubMed were rated as more likely to change practice, but these statistical differences were small in size and unlikely to indicate clinically important differences. It is possible that PubMed contains papers that are more novel, since there would be a greater distribution of systematic reviews in PAIN^+^ due to the filtering processes. While systematic reviews present a synthesis of evidence, clinicians may have been previously exposed to the individual trials and find the results less novel. How new the information is, and how relevant it is, are two different dimensions. Previous studies have highlighted the issues with PubMed’s relative ability to bring out relevant articles when compared to other search engines. A study comparing PubMed to Google Scholar found that Google Scholar retrieved more articles that were relevant than PubMed [12]. Since PubMed and Google Scholar use different search algorithms, they may produce different outputs.

The fact that PAIN^+^ outputs were seen as being more relevant to clinicians may be a positive indicator that the evidence would be relevant and potentially implemented in their practice. This support the premise that targeted evidence support tools may be more effective in effecting change in behaviour. However, since only patient preferences and usefulness ratings measures were examined in this study, not actual behaviour, we cannot confidently extrapolate our findings into behaviour. And the other reason for this could be the fact that we used the default sort order option for the search engines; for PAIN^**+**^ it was “best match” and for PubMed it was “most recent”.

In the current study, likelihood that the studies retrieved by the searches performed by clinicians would change practice was actually very low and it is unlikely that there was a clinically meaningful difference between the two search engines. The scores were less than two in all instances for both search engines. This points out to the fact that there is room for improvement for both search engines. Also, it can also be a window into the literature search skills of the clinician whose search retrieved the article. Either ways it will be premature to make any conclusion based on the ratings of the participants on one article that they retrieved.

We were able to observe that the search strategies used by clinicians were simplistic, often consisting of only the type of pain or type of intervention of interest. Only 29% of the clinicians who were part of the study used Boolean searches and none used delimitators like date or study design to focus on recent or higher quality evidence. This decreased use of advanced tools may result in more irrelevant results which may hamper the search of the clinician with limited time [24]. Our findings are consistent with a previous observational study of search logs clinicians over a 12-month period where Boolean operators were used only 12% of the time [25]. It may be that clinicians know about filtering strategies and do not think they are efficient, or want to peruse a large volume of studies to avoid missing things of interest. The finding that clinicians use cancer pain or non-cancer pain in their search strategy indicates some familiarity with how research literature is categorized as they are important filter terms for pain evidence. It also indicates that clinicians recognize important clinical differences between these two types of pain. The prevalence of searches for cancer pain is not unexpected given that cancer is one of the most common reasons for chronic pain [26]. Also, this could have been driven by the question that was presented to the participants.

A strength of the current study is that we included professionals from multiple disciplines given the inter-disciplinary nature of pain management. However, we were not powered to examine differences between the disciplines, nor were the groups equally represented. Our findings should be considered in light of methodological constraints. Since we limited the number of relevant articles that can be flagged by the clinician to their top three, this affected our ability to calculate total yield or efficiency parameters.

We had some limitations with our outcome measures due to methodological constraints of this study. We acknowledge that it is hard to determine the usefulness of an article just based on the abstract. However, it is reflective of a typical practice among clinicians where they would typically go through the abstract and decide to retrieve the full-text due to time constraints. With the one-page yield: we were limited as the participants were presented with only the first page of the search retrieval which can have up to a maximum of 20 records hitting a ceiling. We did this to manage the search yield as some searches might yield thousands of articles.

Another limitation was that we did not include a wash out period due to the nature of the study environment (online). This may have affected the results of our study because the first search conducted may have affected how the 2nd search was performed, especially if the first search did not provide good results. The randomisation procedures should have minimized the impact this had on our conclusions since any potential carry-over effects were randomly distributed. We recommend future studies to include a wash-out period and take a longitudinal approach to determine the impact or use of these two different search tools over time. A potential source of heterogeneity in the study is the use of different questions for clinicians in the discipline specific scenario and including them in the same analysis. Lastly, the use of the default sort order option for the search engines; could have affected the relevancy ratings for the search engines.

In conclusion, PAIN^+^ and PubMed both were rated as useful in retrieving pain evidence for clinicians across different health disciplines who are involved in pain management. Greater preferences and perceived usefulness of the top 3 retrieved papers was observed for PAIN+, but other dimensions of usefulness did not consistently favor either search engine. Pain^+^ is now freely available for open access use https://www.painpluscpn.ca/.

## Data Availability

All data was available to the authors.

## Appendix 1

### Questions posed to clinicians after each search.Participants will be presented with the first 20 articles retrieved from each Search (1 or 2):

**Participant will check off top 3 relevant papers and rate (without having to read the abstract):**

- **The usefulness of each of the top 3 citations retrieved**

*How useful is this citation?*

**Response:** *1 = not useful at all, 4 = somewhat useful; 7 = very useful*

- **The usefulness of the overall session**
  *How useful is the overall search session?*
  **Response:** *1 = not useful at all, 4 = somewhat useful; 7 = very useful.*

**Participant will pick best paper out of 3 relevant papers and read the abstract to rate:**

- *How would you rate the quality of this paper?*
  **Response:** *Very high, high, moderate, low, very low*
- *Based on the information in this paper, how likely is it that you would change your practice?*
  **Response:** *1 = not likely at all, 4 = somewhat likely, 7 = very likely.*

**After both searches are completed for the multidisciplinary or discipline specific scenario, the participant will be asked the last question:**

- *Which search is better?*

**Response:** *Search 1 or Search 2.*

### Appendix 3

**Table 6:** Search terms and frequency used for discipline specific searches by discipline

Physicians	Reg nurses	Occupational and physical therapists	Psychologists
Neck pain (13) Injection (14) therapy (5) Therapies (3) Effect*(2) Randomized control trial (2) Meta-analysis (2) Clinical trial (2)	Antidepressants (25) Neuropathic pain (37) Management (8) Effect*(4	Low Back Pain (39) Chronic (26) Manipulation (25) Spinal (22) Physiotherapy (22) Physical Therapy (2) Modalities (7) Manipulative therapy (14) Manual Therapy (3) Effectiveness (6) Comparing Outcome (2) Conventional (1) Treatment (10) Results (10) Triggers Point (2) Dry needling (1)	Behavioral therapy (5) Low back pain (4) Chronic back pain (2) Chronic Pain (1) CBT (1)

## Footnotes

1 SPSS Version 22 IBM industries

2 QSR International, Australia
